# A Motivational Model Explaining Performance in Video Games

**DOI:** 10.3389/fpsyg.2020.01510

**Published:** 2020-07-14

**Authors:** Rame Hulaj, Markus B. T. Nyström, Daniel E. Sörman, Christian Backlund, Sebastian Röhlcke, Bert Jonsson

**Affiliations:** ^1^Department of Psychology, Umeå University, Umeå, Sweden; ^2^Department of Human Work Science, Luleå University of Technology, Luleå, Sweden; ^3^Department of Applied Educational Science, Umeå University, Umeå, Sweden

**Keywords:** self-determination theory, basic needs, intrinsic motivation, extrinsic motivation, time on task, video game performance, Dota 2

## Abstract

Esports are a rapidly growing phenomenon and understanding of factors underlying game performance are therefore of great interest. The present study investigated the influence of satisfaction of basic psychological needs (competence, autonomy, and relatedness), type of motivation (amotivation, external regulation, introjected regulation, identified regulation, integrated regulation, and intrinsic motivation), and number of matches played (time on task) on individuals’ performance on a matchmaking rating (MMR) in the video game Defence of the Ancients 2 (Dota 2). Collected data from 315 participants was included in the analyses. A web-based questionnaire was used to collect data and structural equation modelling (SEM) was performed to analyze the data. The results show that perceived competence and autonomy were the only significant predictors of MMR performance beyond matches played. Fulfillment of relatedness, as well as motivational factors, were not found to be predictors of MMR scores. The strong effect of matches played, used as proxy of time on task, emphasize the effect of time and practice as a critical aspect of video-game expertise.

## Introduction

Many people all over the world play video games, independent of gender and across a wide variety of ages. For example, it is estimated that 60% of Americans play video games daily (Entertainment Software Association, 2018), and it is a growing sports phenomenon. E-sport is defined as an organized form of video gaming involving many players either locally or online over the internet. According to Sylvester and Rennie (2017), e-sport, a subcategory of video gaming, is a global activity with no signs of slowing down; the total time spent viewing e-sport is expected to be greater than nine billion hours per year by 2021 (HIS Markit, 2017). According to the Global export market report (Newzoo, 2017), it is estimated that of the 345 million who are involved in e-sport, 45% play, 23% view, and 32% both play and view e-sport. Video gaming is growing, not only as a gaming phenomenon but also as a field of study. Video gaming has been studied from a variety of different areas, such as rehabilitation of gait and balance problems (Ravenek et al., 2016), identifying gaming disorder (Kaptsis et al., 2016), neurological aspect of gaming (Palaus et al., 2017), how gaming affects the brain structure (Brilliant et al., 2019), potential associations between gaming and cognition (Röhlcke et al., 2018; Nuyens et al., 2019), and whether education can be gamified (Kim et al., 2018). Today, video gaming and e-sport are challenging more “traditional” sports in terms of the increasing amount of both recreation players and professional players. However, in comparison to traditional sports, relatively little is known about factors that influence performance.

Indeed, in a recent review, Bányai et al. (2019) argued that few studies have investigated the psychological aspects of e-sports and emphasized that more understanding within this area is essential. For instance, knowledge about gamers’ motivational patterns can be helpful when trying to foresee negative consequences such as gaming disorders, which according to the World Health Organization (WHO),^1^ is classified as a mental health problem. WHO defines gaming disorders as when “people who partake in gaming should be alert to the amount of time they spend on gaming activities, particularly when it is to the exclusion of other daily activities, as well as to any changes in their physical or psychological health and social functioning that could be attributed to their pattern of gaming behavior^2^”. There are, however, also possible benefits of video gaming. It has been suggested that video gaming can improve cognitive processes (e.g., improved attention control and processing speed; Nuyens et al., 2019) and postpone cognitive decline (Griffiths et al., 2013).

Previous studies have identified specific characteristics underlying gaming motivations (Vorderer, 2000; Vorderer et al., 2003; Sherry et al., 2006; Yee, 2006a, b; Greenberg et al., 2010; Demetrovics et al., 2011). For example, Vorderer (2000) and Vorderer et al. (2003) argue that interactivity and competition are two of those characteristics, with the former being related to communication and cooperation, whereas the latter is related to the possibility to compare themselves with other players. In another study, it was found that, for college students, challenges, diversions, and competition were the strongest types of motivation (Greenberg et al., 2010).

Röhlcke et al. (2018) investigated the predictive ability of several factors (i.e., number of matches played, working memory capacity, grit, fluid intelligence, age, and education) for performance in the multiplayer video game “Defense of the Ancients 2” (Dota 2). The study showed that the number of matches played (proxy for time on task) was the strongest predictor of performance, but no effect of cognition was obtained, which is in contrast to other findings (e.g., Nuyens et al., 2019).

The effect of the number of matched played (time on task) is in line with the studies showing that learning and performance progress when time spent on the task increases. In a study of 15-year-old students’ homework, Wagner et al. (2008) found a positive but weak relationship between the amount of time students work at home and scholastic achievements. An argument was found valid also for the Programme for International Student Assessment data on student homework; hence, the frequency of homework in mathematics was predictive of students’ mathematical performance (Trautwein, 2007) [see also Cooper (1989) and Smith (1990) for similar results]. Findings indicate that time on task is a critical factor for improvements and potentially also for video gaming and e-sport performance–the more you play, the better you perform. Similarly, in a review by Baker and Young (2014), the authors confirmed the importance of practice in more traditional sports (e.g., football).

**FIGURE 1:**
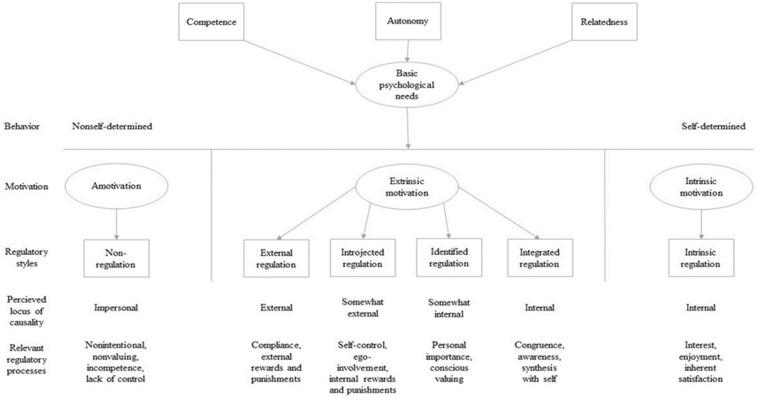
A taxonomy of human motivation, presented on a continuum, ranging from no motivation to controlled and a more autonomous form of motivation. The satisfaction of basic psychological needs forms a more autonomous motivation. The figure add the basic psychology needs to the Ryan and Deci (2000a,b) taxonomy of human motivation.

Why and to what extent time on task is critical for performance are a fundamental question that for the fast-growing e-sport is lacking a clear answer. Gaining knowledge about factors that have an impact on time spent playing is not only interesting from a performance perspective, it may, to some extent, also explain why e-sports are a growing phenomenon and why many people choose to spend a lot of time (or not) on playing e-sports. According to Deci and Ryan (2000), in the self-determination theory (SDT), the satisfaction of the basic psychological needs relatedness, competence, and autonomy are assumed to guide the individual toward a more vital, competent, and socially integrated behavior; especially autonomy and competence plays a significant role in facilitating an individual’s intrinsic motivation to perform an activity (Uysal and Yildirim, 2016). These basic psychological needs are considered essential to one’s sense of well-being and psychological growth. *Competence* refers to the propensity to strive toward mastery and being optimally challenged. When the *autonomy* need is fulfilled, the individual is left with a sense of control and freedom when performing a specific activity. *Relatedness* refers to having a sense of belongingness and a meaningful connection to others (Deci and Ryan, 2000; Ryan and Deci, 2000b; Uysal and Yildirim, 2016). Motivation, according to SDT, is viewed along a continuum ranging from *amotivation*, *extrinsic motivation*, to *intrinsic motivation.* Amotivation is when one is entirely unmotivated because the activity does not generate feelings of competence, does not bring any value, and does not feel worthwhile. Extrinsic motivation refers to the forms of regulation that underlie actions that individuals perform as means to get to the end, whereas intrinsic motivation is characterized by a genuine interest and passion for an activity (Figure 1; Ryan and Deci, 2000a,b).

Extrinsic motivation can be split into four subcategories, depending on whether the motivation type is controlled or autonomous (Deci and Ryan, 2008). These include *external regulation*, *introjected regulation*, *identified regulation*, and *integrated regulation*. External regulation, which is the least autonomous form of motivation, can occur when it is primarily external factors that motivate the individual to perform an activity. These external factors could be either in the form of rewards or punishments. *Introjected regulation* describes a type of motivation that arises when an individual does something to avoid guilt or anxiety or to boost their ego by demonstrating their abilities to maintain or increase their self-esteem. Identified regulation refers to behavior that is associated with greater feelings of freedom and volition because their behavior is more congruent with their individual goals and personal identities. They understand their behavior as a reflection of themselves. Integrated regulation, the most autonomous form of extrinsic motivation, occurs when one identifies with the task, values, and needs that the task brings. The reasons for engaging in an activity are further assimilated to the self and are thus autonomous. However, the individual is still extrinsically motivated (rather than intrinsically motivated) as they engage in the activity based on presumed outcomes, rather than for an inherent passion or interest for the activity (Ryan and Deci, 2000a, b).

Furthermore, research suggests that intrinsic motivation has been associated with positive outcomes, such as performance, concentration, persistence, and well-being, as assessed across different activities and situations (Ryan and Deci, 2000a; Gillet et al., 2009). For example, a previous study found a positive correlation between self-determined motivation and performance (i.e., the ratio between the number of victories and the number of matches played), suggesting that self-determined motivation may influence performance among tennis players (Gillet et al., 2009).

With respect to e-sport (as well as for more traditional sports), previous studies have shown that the fulfillment of basic psychological needs is associated with players’ willingness to continue playing (e.g., Ryan et al., 2006), which in turn have a large impact on motivation and a positive effect on the development of more intrinsically regulated motivation (Ryan and Deci, 2000a, b). The satisfaction of basic psychological needs may offer an explanation to why people play video games in the first place; the gaming simply enhances the enjoyment and thus provides the satisfaction of their basic psychological needs (Ryan et al., 2006; Przybylski et al., 2009; Tamborini et al., 2010; Rogers, 2017). Potentially, this satisfaction also predicts how likely a player is to continue playing the game in the future (Ryan et al., 2006). However, different types of video games seem to satisfy different basic psychological needs. For example, a recent study by Rogers (2017), who investigated the motivational pull of video games, found that social elements within the games lead to feelings of relatedness, and games consisting of flexible rules encouraged feelings of competence.

As noted above, Röhlcke et al. (2018) pointed out matches played (time on task) as a strong predictor of performance in Dota 2. If matches played are related to performance to the extent suggested by Röhlcke et al. (2018), then it seems necessary to include this factor when investigating the role of time on task on video game performance. In addition, considering the few studies that have investigated the importance of matches played for video game performance (and Dota 2 specifically), there is also a need to replicate the findings by Röhlcke et al. (2018) to be able to establish the findings. The authors did not, however, investigate the effects of different forms of motivation and satisfaction of basic psychological needs on performance in Dota 2. The conjunction of basic psychological needs (autonomy, competence, relatedness), different types of motivation (amotivation, external regulation, introjected regulation, identified regulation, integrated regulation, intrinsic motivation), and matches played (time on task) are therefore critical variables in the present study.

This brief introduction shows that e-sports have established itself all over the world, with certain games having thousands of players (such as Dota 2); it gains more media and public interest. Today, e-sports have become an industry challenging more “traditional” sports. In some e-sports, such as Dota 2, professional players are common. However, compared to more “traditional sports” (team sports and individual sports), relatively little is known about factors that influence game performance, such as time on task. Even less is known to what extent psychological factors such as basic psychological need and motivation play a role. More knowledge about factors that have an impact on time spent playing and competing in e-sports is not only interesting from a performance perspective, but it may also, to some extent, explain why e-sports is a growing phenomenon and why many people choose to spend a lot of time (or not) on playing e-sports.

### Aim

The main argument for the present study is to understand to what extent time on task (number of matched played), basic psychological needs, and motivational factors influence players’ online gaming performance. Information that, by extension, can be used to foresee player behavior that potentially can evolve into inadequate social and interpersonal behavior such as gaming withdrawal (Kaptsis et al., 2016). In addition, there is, to our knowledge, no study that has included basic psychology needs, types of motivation, and matches played (time on task) as predictors of video game performance. The aim of the present study was therefore to investigate whether and to what extent the satisfaction of basic psychological needs, different types of motivation, and matches played are associated with the video gaming performance.

## Materials and Methods

### Video Game

In the present study, the real-time strategy (RTS) game Dota 2 was used as the video game of interest. Dota 2 is a free-to-play game and is available on personal computers. The game was developed by Valve Corporation (first released in 2013) and is regarded as a multiplayer online battle and is one of the most successful games with respect to the price money of competitive gaming. Dota 2 had the highest accumulated prize pool distributed among professional e-sport (electronic sports) players in 2019 (E-sports earnings, 2019). The game also has the highest player count for any game on the Valve Corporation platform, and in February 2019, Dota 2 had an average of 475,747 active players playing the game every hour of the day, and there were a total of 11.3 million players registered (Steam, 2019). In Dota 2, two teams of five players compete against each other. The main objective is to destroy the enemy base. Each player controls a hero with unique abilities and characteristics, which are improved throughout the game by leveling up and obtaining different equipment (e.g., armor, arms gloves, etc.) for the hero. Dota 2, currently consists of 117 heroes, 152 items, and more than 480 distinctive spells such as jumping high, flying, becoming invisible, and so on (Röhlcke et al., 2018; Dota 2 Gamepedia, 2019). For the present study, two measures from Dota 2 were used: Matchmaking rating (MMR) and Matches played (see detailed description below).

### Procedure

Participants were recruited through advertisements in Dota-specific internet communities (e.g., Reddit) and through email. The email was sent out to participants who had previously participated in a Dota 2 study and had approved to be contacted again. Contact information for new registers was obtained as part of the test battery. The response rate could not be calculated because we could not register how many saw or read the advertisement. In the advertisement, it was emphasized that the study aimed to investigate the relationship between performance, personality, and motivation in Dota 2. If interested to participate, they were asked to fill out the online questionnaire. To be included in the present study, participants needed to have played at least 110 ranked games (see under the description of MMR below) of Dota 2 and a minimum of 10 games during the past month. These selection criteria are similar to what has been used previously (see Röhlcke et al., 2018). The questionnaire used to collect data was distributed using Google forms. Participants were first presented with information about the project, including information related to the fact that participation was voluntary and that participants had the right to cancel their participation at any time. This information was followed by specific instructions and a letter of consent. After providing their informed consent, participants provided their background information, such as age, gender, and highest education level, after which they answered Dota-specific questions and questions about their motivation for playing the game. The survey took approximately 25 min to complete.

### Participants

A total of 329 Dota 2 players agreed to participate in the study. An initial screening revealed 14 statistical outliers according to the three-interquartile-range rule, which were consequently removed from the analysis. Thus, the final sample consisted of 315 participants. Among them were 299 males (94.9%) and 13 females (4.1%); 3 participants preferred not to state their gender (1%). The mean age of the participants was 23.32 years (SD = 4.52 years), and participants were recruited from 60 different countries. The level of education attained by the participants in the sample included primary school (4.8%), junior high school (3.0%), high school (23.2%), trade/technical/vocational training (3.5%), some college/university credits (18.4%), professional degree (2.2%), bachelor’s degree (35.9%), associate degree (2.9%), master’s degree (7.9%), and doctorate degree (1%).

### Measures

#### MMR

A player’s MMR score represents performance on Dota 2. In ranked games, an algorithm is used to calculate how many MMRs players win or lose after the game is played. If players win a game, they receive a point between +25 and +30, and if players lose, they receive a point between −25 and −30. More MMRs points are received if the opponents are considered to be overall slightly better, and less if the opponents are considered to be slightly worse. This system places players in games with similarly skilled players. As such, higher MMR scores are indicative of a more highly skilled player, whereas a lower MMR indicates that a player is less skilled (Röhlcke et al., 2018; Dota 2 Gamepedia, 2019). In this study, MMR scores were self-reported by the participants in the questionnaire. Participants are able to retrieve their MMR scores within the game. See Röhlcke et al. (2018) for a full explanation. The mean MMR in this sample was 3359.66 (SD = 1294.17, min = 35, max = 7274). MMR was found to be normally distributed with skewness of 0.0 and kurtosis of −0.2. A threshold of 2 for skewness and 7 for kurtosis have been suggested in the literature (e.g., Finney and DiStefano, 2006).

#### Matches Played

Participants reported the total number of games played in Dota 2. This information was available for the participants within the game and was thus used as a proxy of “time on task.” The average number of matches played by the participants was 3,552.9 (SD = 2,551.9), with skewness of 1.6 and kurtosis of 2.8, which is acceptable for normally distributed data (Finney and DiStefano, 2006). We do not know the time period taken to reach the number of matches played, but the large spread in data (as indicated by the SD) increases the likelihood of finding plausible effects of matches played on MMR.

#### Motivation

The Gaming Motivation Scale (GAMS) was used to determine motivational characteristics. The GAMS includes the following factors: (1) amotivation, (2) external regulation, (3) introjected regulation, (4) identified regulation, (5) integrated regulation, and (6) intrinsic motivation. Factors 2 to 5 are each related to extrinsic motivation. Three items represent each of the factors in the questionnaire. Some of them were adjusted slightly to fit the Dota 2 game better. One item was also added to target whether players played the game with the aim of gaining MMR points. For each item, the respondents rated their level of agreement with each using a seven-point Likert scale, with responses ranging from 1 (“do not agree at all”) to 7 (“very strongly agree”). Thus, the maximum score for each factor was 21 (three items × seven-point Likert scale). Questions were framed using the following stem: “Why do you play Dota 2?” “Rate your agreement with the following statements.” Participants then responded to questions related to intrinsic motivation (e.g., “because it is stimulating to play”), integrated regulation (e.g., “because it is an extension of me”), identified regulation (e.g., “because it is a good way to develop important aspects of myself”), introjected regulation (e.g., “because I feel that I must play regularly”), external regulation (e.g., “for the prestige of being a good player”), and amotivation (e.g., “it is not clear anymore; I sometimes ask myself if it is good for me”). In the present study, skewness ranged from −0.4 to 0.6 and kurtosis from −0.8 to 0.1 for the variables included in GAMS, which demonstrates normally distributed data. For each factor included in GAMS, a mean score was calculated. In a study performed by Lafrenière et al. (2012), GAMS had a Cronbach’s α value between 0.75 and 0.89. In this study, the Cronbach’s α values were between 0.52 and 0.88. Peterson (1994) suggests that an acceptable Cronbach’s α is between 0.50 and 0.60 for preliminary research, whereas for basic and applied research, the Cronbach’s α should be at least 0.70. Nunnally (1978) also suggests an acceptable Cronbach’s α of 0.70.

#### Basic Psychological Needs

Player Experience of Need Satisfaction (PENS) was used to measure participant satisfaction related to their basic psychological needs (Ryan et al., 2006). Three items per subscale (competence, autonomy, and relatedness) were used as indicators of each basic need. This scale uses a seven-point Likert scale with responses ranging from 1 (“do not agree at all”) to 7 (“very strongly agree”). The questions were framed with the following stem: “Reflect on your play experiences with Dota 2 and rate your agreement with the following statements.” Each question then reflected either competence (e.g., “I feel competent at the game”), autonomy (e.g., “I experienced a lot of freedom in the game”), or relatedness (e.g., “I find the relationships I form in this game important”). Skewness ranged from −0.9 to −0.3 and kurtosis from −0.4 to 0.4, thus demonstrating a normally distributed data. A mean score was calculated for each basic psychological need included in PENS. In a study by Lafrenière et al. (2012), it was concluded that an acceptable range of Cronbach’s α values for PENS is between 0.72 and 0.80. Cronbach’s α values in the present study were between 0.74 and 0.83, indicating acceptable internal consistency (Nunnally, 1978; Peterson, 1994).

### Statistical Analyses

First, the descriptive information of the study sample was summarized. Then, zero-order correlations were conducted between all the variables included in the analyses. For descriptive information and correlation analysis, the mean scores for each basic psychological and motivation factor were used. Next, structural equation modeling (SEM) was used to investigate the effects of basic psychological needs, motivation, and matches played on MMR (the dependent variable). For each basic psychological need and motivation factor, single items were used as indicators of the latent variable representing each factor/construct. In the model, type of motivation (amotivation, external regulation, introjected regulation, identified regulation, integrated regulation, intrinsic motivation) was also assumed to be predicted by basic psychological needs (competence, autonomy, and relatedness). Three fit indices were used to evaluate the model, including the comparative fit index (CFI), the root mean square error of approximation (RMSEA), and χ^2^ divided by degrees of freedom. To attain an acceptable fit for CFI, the value must be equal to or greater than 0.95 (Browne and Cudeck, 1989). RMSEA values need to be equal to or less than 0.06 to attain a good model fit and 0.08 for a reasonable fit (Browne and Cudeck, 1989; Hu and Bentler, 1999). For normed χ^2^ results, the suggested threshold values range from 2.0 (Tabachnick and Fidell, 2007) to 5.0 (Wheaton et al., 1977) in the literature. The data were analyzed using SPSS (IBM Corporation, Armonk, NY, United States) and AMOS 26 (Arbuckle, 2016). Initial analyses revealed that the demographic data were non-significant in relation to the MMR and were therefore excluded from further analysis.

## Results

Descriptive data of variables included in the analyses are presented in Table 1. As can be seen, for both skewness (range = −0.9 to 1.6) and kurtosis (range = −1.2 to 2.8), the variables included indicated normally distributed data.

**TABLE 1:** Descriptive data of variables included in the analyses.

Variable	Mean	*SD*	Skewness^a^	Kurtosis^b^
MMR	3359.7	1294.2	0.0	–0.2
Matches Played	3552.9	2551.9	1.6	2.8
Amotivation	3.7	1.9	0.1	–1.2
Intrinsic motivation	5.4	1.0	–0.3	–0.3
**Extrinsic motivation**				
Identified regulation	4.1	1.4	–0.2	–0.5
External regulation	4.3	1.2	–0.4	0.1
Integrated regulation	3.9	1.5	–0.1	–0.8
Introjected regulation	3.0	1.5	0.6	–0.6
**Basic Psychological Needs**				
Competence	5.2	1.0	–0.3	–0.4
Autonomy	6.0	0.9	–0.9	0.4
Relatedness	4.2	1.4	–0.3	–0.4

*^a^Std. Error 0.137, ^b^Std. Error 0.274.*

Zero-order correlations between the variables are presented in Table 2. As can be seen, MMR score was positively associated with matches played, integrated regulation, competence, autonomy, and relatedness. Matches played, highly correlated with MMR, were also associated with amotivation, integrated regulation, and competence. In addition, all factors related to motivation and basic psychological needs were, to a large extent, related to each other. Next, to further investigate the complexity between factors, SEM analyses were performed.

**TABLE 2:** Correlations between variables used in structural equation model.

	1	2	3	4	5	6	7	8	9	10	11
(1) MMR	–										
(2) Matches Played	0.59**	–									
(3) Amotivation	0.10	0.14*	–								
(4) Intrinsic motivation	0.02	–0.06	−0.13*	–							
(5) Identified regulation	0.05	0.00	–0.01	0.39**	–						
(6) External regulation	0.07	0.08	0.17**	0.24**	0.30**	–					
(7) Integrated regulation	0.18**	0.12*	0.01	0.39**	0.64**	0.35**	–				
(8) Introjected regulation	–0.02	–0.01	0.27**	0.16**	0.43**	0.48**	0.53**	–			
(9) Competence	0.44**	0.30**	–0.04	0.22**	0.20**	0.23**	0.31**	0.10	–		
(10) Autonomy	0.18**	0.11	−0.22**	0.37**	0.28**	0.12*	0.21**	0.05	0.21**	–	
(11) Relatedness	0.12*	–0.01	–0.03	0.33**	0.39**	0.09	0.30**	0.11	0.16**	0.28**	–

*p < 0.05, **p < 0.01, ***p < 0.001.

**FIGURE 2:**
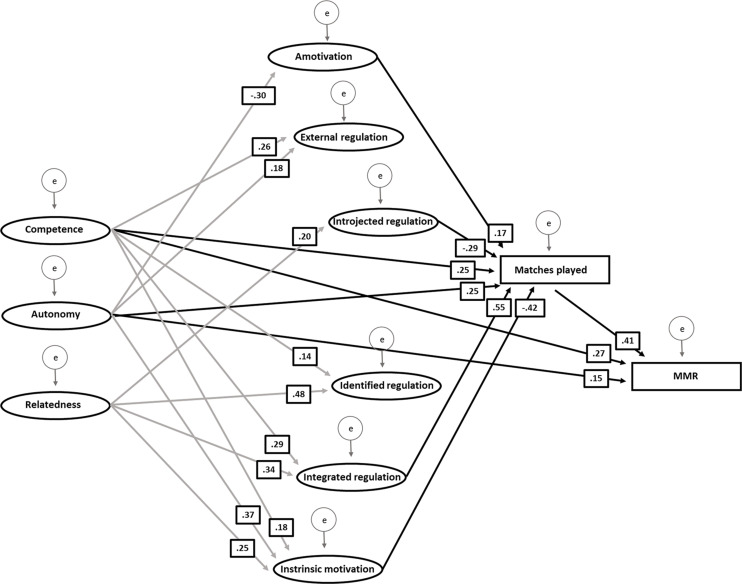
Structural equation model illustrating significant paths together with standardized regression weights between basic psychological needs (competence, autonomy, and relatedness), motivation types (intrinsic motivation, identified regulation, external regulation, integrated regulation, introjected regulation, amotivation), matches played, and matchmaking rating (MMR) in Dota 2. Black arrows show significant relationships on MMR and matches played. Gray arrows show significant relationships between basic psychological needs and the type of motivation. Latent variables are represented by ovals, and all manifest variables are represented by rectangles.

The results of standardized and unstandardized β weights from the SEM analysis accompanied by standard errors and *p* values can be seen in Table 3. In the SEM analysis, latent variables were used to represent each basic psychological need and motivation factor. The factor loadings for the latent constructs ranged from 0.36 to 0.88 (mean = 0.70, SD = 0.15), and 43% of the factors loadings were greater than 0.80. The model indicated acceptable fit with regard to RMSEA (0.065, PCLOSE < 0.001) and normed χ^2^ (χ^2^/*df* = 2.320, *p* < 0.001), although poor with regard to CFI (0.880). As can be seen, matches played are a strong predictor of performance (MMR). Among the other predictors included in the model, the basic psychological needs autonomy and competence also reached statistical significance (Table 3 and Figure 2). The remaining basic psychological need, relatedness, and all motivation factors are non-significant predictors of performance. Matches played, which is a strong predictor of MMR score, was similarly as for MMR score positively predicted by basic psychological need factors autonomy and competence. Among factors related to motivation, integrated regulation and amotivation positively related to matches played, whereas intrinsic motivation and introjected regulation are negatively associated with the number of matches played. These factors were, in turn, significantly predicted by many of the basic psychological need factors (Table 3), which demonstrates the complexity of the results. The model explained 27% (*R*^2^ = 0.27) of the variance of matches played, and 48% (*R*^2^ = 0.48) of the variance of MMR.

**TABLE 3:** Standardized regression weights of the predictor variables used in the structural equation model with matchmaking rating as the dependent variable.

	β	*B*	*S.E.*	*P*
Matches played → MMR	0.41	0.21	0.03	<**0.001**
Amotivation → MMR	0.08	59.49	46.78	0.203
Intrinsic motivation → MMR	–0.19	–397.04	302.69	0.189
Identified regulation → MMR	–0.21	–215.04	184.31	0.243
External regulation → MMR	0.13	153.28	101.80	0.132
Integrated regulation → MMR	0.27	229.72	142.78	0.108
Introjected regulation → MMR	–0.16	–158.65	98.88	0.109
Autonomy → MMR	0.15	286.00	139.97	**0.041**
Relatedness → MMR	0.12	105.25	66.87	0.116
Competence → MMR	0.27	345.27	77.04	<**0.001**
Amotivation → Matches played	0.17	251.93	118.29	**0.033**
Intrinsic motivation → Matches played	–0.42	–1728.09	761.14	**0.023**
Identified regulation → Matches played	–0.24	–493.46	455.55	0.279
External regulation → Matches played	0.18	410.36	251.57	0.103
Integrated regulation → Matches played	0.55	917.60	339.95	**0.007**
Introjected regulation → Matches played	–0.29	–579.78	241.54	**0.016**
Autonomy → Matches played	0.25	935.15	337.21	**0.006**
Relatedness → Matches played	–0.01	–11.88	167.82	0.944
Competence → Matches played	0.25	630.83	191.49	<**0.001**
Competence → Amotivation	0.02	0.03	0.11	0.762
Relatedness → Amotivation	0.09	0.11	0.08	0.180
Autonomy → Amotivation	–0.30	–0.73	0.19	<**0.001**
Competence → Intrinsic motivation	0.18	0.11	0.05	**0.016**
Autonomy → Intrinsic motivation	0.37	0.34	0.08	<**0.001**
Relatedness → Intrinsic motivation	0.25	0.11	0.03	**0.001**
Competence → Identified regulation	0.14	0.17	0.09	**0.049**
Autonomy → Identified regulation	0.14	0.26	0.14	0.061
Relatedness → Identified regulation	0.48	0.41	0.07	<**0.001**
Competence → External regulation	0.26	0.29	0.08	<**0.001**
Autonomy → External regulation	0.18	0.31	0.13	**0.019**
Relatedness → External regulation	–0.06	–0.04	0.06	0.444
Competence → Integrated regulation	0.29	0.45	0.10	<**0.001**
Autonomy → Integrated regulation	0.05	0.11	0.15	0.476
Relatedness → Integrated regulation	0.34	0.35	0.07	<**0.001**
Competence → Introjected regulation	0.08	0.10	0.09	0.277
Autonomy → Introjected regulation	–0.09	–0.16	0.14	0.246
Relatedness → Introjected regulation	0.20	0.17	0.06	**0.008**

*β = Standardized regression weight, B = Unstandardized regression weight, S.E. = Standardized error of B, MMR = Matchmaking rating.*

In addition to analyses of direct effects, we also investigated possible indirect (mediating) effects. Results showed that there were a significant indirect effects of matches played on the relationship between competence and MMR (β = 0.175, *p* = 0.030), but not for relatedness (β = *−*0.127, *p* = 0.194) or autonomy (β = *−*0.032, *p* = 0.760). Thus, for competence, the number of matches played to some extent can explain the relationship with MMR. For autonomy, however, non-significant effects suggest that the relationship between autonomy and MMR is direct and is not mediated by the number of matches played. There was also a significant indirect effect found of matches played on the relationship between intrinsic motivation and MMR (β = *−*0.170, *p* = 0.049). Thus, the result shows that the relationship between intrinsic motivation and MMR is not only direct but also mediated by matches played (time on task). However, matches played did not mediate any effects of introjected regulation (β = *−*0.118 *p* = 0.104), amotivation (β = 0.071, *p* = 0.082), integrated regulation (β = 0.227, *p* = 0.078), external regulation (β = 0.073, *p* = 0.133), or identified regulation (β = *−*0.098, *p* = 0.349) on MMR.

**FIGURE 3:**
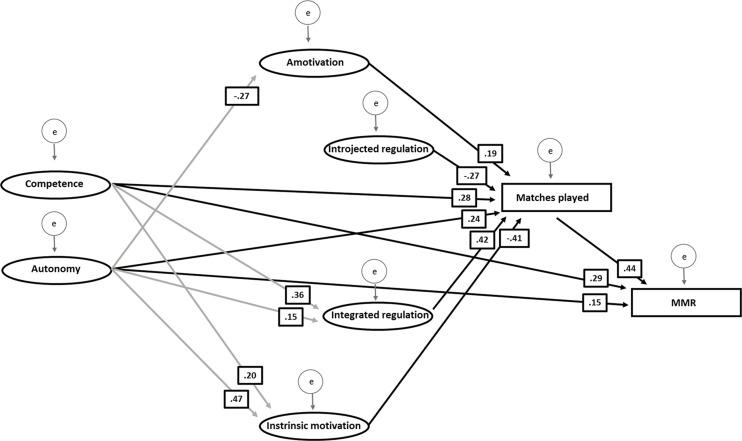
A trimmed structural equation model illustrating significant paths together with standardized regression weights between basic psychological needs (competence, autonomy), motivation types (intrinsic motivation, integrated regulation, introjected regulation, amotivation), matches played, and matchmaking rating (MMR) in Dota 2. Black arrows show significant relationships on MMR and matches played. Gray arrows show significant relationships between basic psychological needs and type of motivation. Latent variables are represented by ovals, and all manifest variables are represented by rectangles.

Because relatedness, identified regulation, and external regulation were not significant as predictors of either matches played or MMR, they were removed for a final trimmed structural model. For this model, all model fits were acceptable (CFI = 0.960, RMSEA = 0.047, PCLOSE = 0.682, χ^2^/*df* = 1.691, *p* < 0.001). As expected, all significant paths toward matches played and MMR remained significant in the trimmed model (Table 4 and Figure 3). The only difference from the main analysis is that autonomy became a significant predictor of integrated regulation. However, this effect was very small.

**TABLE 4:** Standardized regression weights of the predictor variables used in the trimmed structural equation model with matchmaking rating as the dependent variable.

	β	*B*	*S.E.*	*P*
Matches played → MMR	0.44	0.22	0.03	<**0.001**
Amotivation → MMR	0.11	82.08	42.81	0.055
Intrinsic motivation → MMR	–0.12	–254.94	257.98	0.323
Integrated regulation → MMR	0.14	113.47	99.86	0.256
Introjected regulation → MMR	–0.13	–135.16	84.97	0.112
Autonomy → MMR	0.15	281.57	133.72	**0.035**
Competence → MMR	0.29	375.36	72.51	<**0.001**
Amotivation → Matches played	0.19	294.13	107.12	**0.006**
Intrinsic motivation → Matches played	–0.41	–1659.36	668.46	**0.013**
Integrated regulation → Matches played	0.42	662.90	250.98	**0.008**
Introjected regulation → Matches played	–0.27	–548.21	210.37	**0.009**
Autonomy → Matches played	0.24	889.25	332.33	**0.007**
Competence → Matches played	0.28	713.92	175.71	<**0.001**
Competence → Amotivation	0.03	0.05	0.11	0.627
Autonomy → Amotivation	–0.27	–0.66	0.17	<**0.001**
Competence → Intrinsic motivation	0.20	0.13	0.05	**0.009**
Autonomy → Intrinsic motivation	0.47	0.43	0.08	<**0.001**
Competence → Integrated regulation	0.36	0.53	0.11	<**0.001**
Autonomy → Integrated regulation	0.15	0.36	0.16	**0.022**
Competence → Introjected regulation	0.10	0.13	0.09	0.149
Autonomy → Introjected regulation	–0.03	–0.05	0.13	0.680

*β = Standardized regression weight, B = Unstandardized regression weight, S.E. = Standardized error of B, MMR = Matchmaking rating.*

## Discussion

The aim of the present study was to examine whether and to what extent number of matches played (time on task), basic psychological needs, and motivational factors predict performance (MMR) in Dota 2. The results showed that basic psychological need competence and autonomy, but not relatedness, were significant predictors of MMR. This finding corresponds, in part, with the findings of Van Nuland et al. (2012), who found that competence was directly associated with persistence and performance. However, no effects were found for motivational factors (direct or indirect) on MMR. In line with the findings of Röhlcke et al. (2018), matches played were a strong predictor of MMR, and it was thus justified to include this factor in the model. A final trimmed structural equation model, in which non-significant predictors from the main analysis were removed, confirmed the overall findings from the main model.

The need competence, which is related to the strive toward mastery and challenges, was a significant predictor of performance. This is in line with the results from Rogers et al. (2017), who recently suggested that games with flexible rules boost feelings of competence, and has previously been linked to performance within traditional sports such as football (see, e.g., Fransen et al., 2018). In this study, we found a similar pattern, which suggests that feeling of competence is a factor that can contribute to player performance. Thus, also within the context of video gaming, it seems reasonable to suggest that competence-promoting strategies are something to strive for to promote performance.

We also found autonomy to be a factor related to performance. Thus, the importance of a sense of control and freedom plays a role for performance in Dota 2. However, the effect was rather small. This was perhaps somewhat surprising considering the general need for autonomy for optimal functioning found in earlier studies (see, e.g., Deci and Ryan, 2004). However, results are in line with previous findings that have reported a more robust relationship between competence and performance than between autonomy and performance (e.g., Cerasoli et al., 2016). The small effect could potentially be explained by the fact that Dota 2 is a team-based game. Even though more training (more matches played) improves performance, the individual player is always dependent on his/her team during a game and therefore, potentially, does not experience a sense of increased autonomy when performance improves as a function of more matches played.

As noted, relatedness was not a significant predictor of MMR. A previous study found that socialization factors were a significantly greater motivator for women who played video games than for men (Sun, 2017). In part, this could explain the non-significant relation between relatedness and MMR in the present study; hence, only 5% of the participants were women. If the population would have been more heterogenic, perhaps relatedness would have been significantly related to MMR.

The non-significant effects for intrinsic motivation on MMR were somewhat surprising and inconsistent with previous research, which have reported intrinsic motivation to be a predictor of performances (Deci and Ryan, 2000; Ryan and Deci, 2000a; Gillet et al., 2009). A possible explanation for this could be that Dota 2 is an externally reward-based game with incentives that have a direct link to performance (i.e., MMR scores are always visible and are a direct reflection of performance). Previous studies have suggested that incentives that have a more direct link to performance do not facilitate intrinsic motivation (Cerasoli et al., 2016). Through the in-game feedback (performance direct incentives), intrinsic motivation could become less vital and extrinsically more vital (Mekler et al., 2017). This argumentation is in line with more recent results, which indicate that more direct incentives do not impact on intrinsic motivation but do have a positive impact on performance (Greene, 2018).

There are, of course, several other possible explanations to the non-significant effects of intrinsic motivation. Players may have explicit motives to play video games, such as to enhance their skill development or to experience various social aspects of the game (Demetrovics et al., 2011; Hamari et al., 2015; Wu et al., 2016). It is also possible that these findings reflect subconscious selection effects. Some players may select games that fit their personality traits (Graham and Gosling, 2013) and satisfy their needs in various ways, whereas some games emphasize social elements, which can lead to feelings of relatedness (Johnson and Gardner, 2010; Rogers, 2017). Another potential explanation for the non-significant relationship between performance and intrinsic motivation is the interaction between personality trait and type of motivation. Previous studies have shown that, although intrinsic motivation is considered an aspect that spurs creativity, it does not work in isolation, but only in combination with certain personality traits (i.e., openness; Prabhu et al., 2008; Agnoli et al., 2015, 2018). This could be an explanation for the non-significant relationship between intrinsic motivation and performance, but because we did not control for personality type in this study, this is only speculation. This highlights the need for future studies to also consider personality when investigating the role of motivational type for performance in a video game context.

Similar to the finding that intrinsic motivation was not related to MMR, it did not have a positive impact on matches played. In fact, there was a negative association between intrinsic motivation and matches played. Possible explanations for this finding are most likely similar to those discussed previously in relation to MMR, related to in-game characteristics. Hence, as the players level up in the game, they are rewarded, suggesting that extrinsic motivation becomes more vital (Mekler et al., 2017).

It should, however, be noted that integrated regulation, a factor underlying extrinsic motivation, which occurs when one identifies with the task and the requirements of the game, was a strong predictor of matches played. Although integrated regulation shares qualities with intrinsic motivation, it is driven by extrinsic goals, such as the in-game incentives, which in turn suggests why integrated regulation was found to be a significant predictor. Introjected regulation, on the other hand, which also is regarded as part of extrinsic motivation, was negatively associated with matches played (time on task). Introjected regulation is related to an individual’s motivation to do things not solely because he wants to, but to avoid guilt and for a sense of obligation and to protect the individual’s ego. Because Dota 2 is a team-based game, which perhaps would support a positive association, this was somewhat surprising. Although speculative, as teammates relatively often change and are easily replaced in Dota 2, and as the player is not forced as an individual player to play with a certain team, it is plausible that the influence of teammates on introjected regulation is less than it would be if teammates where more static and more difficult to replace as in more traditional sports (e.g., football). Still, Dota 2 is a team game where the individual’s mistakes become visible and its consequences on the team’s performance apparent, it could be perceived as threatening to the individual’s ego. If the threat of being revealed as the “weak link” becomes too great, it could perhaps explain why introjected regulation has a negative relationship with matches played. Based on the assumption that the protection of the individual’s ego is a relatively central reason for the negative relationship between introjected regulation and matches played (in the present context), this could partly explain the different direction of the relation between introjected regulation and matches played (negative) and integrated regulation and matches played (positive). Because integrated regulation does not place as much focus on protecting the ego, the risk of being exposed as the “weak link” may not be perceived as threatening and thus does not affect motivation to play to the same extent. However, it should be mentioned that previous studies have concluded that more intrinsic and extrinsic motivational tendencies do not rule out one another, but tend to rather dynamically coexist in effecting creativity (Agnoli et al., 2018). This could also be the case when it comes to performance within a video game context. The differences between integrated regulation and introjected regulation in relation to matches played (time on task) illustrate the importance of investigating them separately and not as part of the same construct (extrinsic motivation) in the context of e-sports.

A further surprising finding is the positive relationship between amotivation and matches played. It may seem odd that one plays even more when at the same time unmotivated because the game does not bring feelings of competence, any value, or worthwhile. This result was a major surprise, and there is no straightforward explanation for this finding. One account for this result could potentially be that playing Dota 2, as well as playing other video games, can develop into a regular habit or routine to, for instance, kill time during downtime. Thus, playing Dota 2 can also be driven by factors not related to competence, value, or worthwhile. Instead, playing can be a way to have something to do during periods. However, this is highly speculative, and we do not know if these findings are related to sample characteristics or are specific for Dota 2 players, or video gamers as a whole. Future studies should examine this further.

In this study, we also investigated if matches played could act as a mediator of the relationship between motivational factors and MMR. However, the only significant indirect effect of matches played was found for the relationship between intrinsic motivation and MMR, which was negative. Thus, no motivational factors, not even through other pathways, had any positive effect on MMR. With regard to basic psychological needs, the mediating effect of matches played on the relationship between competence and MMR indicates that competence, in contrast to autonomy, also develops alongside with the number of matches played. It seems fairly reasonable to assume that playing more matches increases the sense of competence, given that the team also wins a fair amount of games.

Although not the primary focus of this study, the results indicated that all three basic psychological needs, competence, autonomy, and relatedness, were significantly predictive of intrinsic motivation. The results from the present study also indicated a negative relationship between autonomy and amotivation. This supports previous research suggesting that all three basic psychological needs are important factors for intrinsic motivation (Uysal and Yildirim, 2016). We cannot determine whether this finding is sample-specific or specific to Dota 2, and therefore, further investigations are needed. It should, however, be noted that previous studies have indicated similar findings (Mitchell et al., 2020).

The present study has some fundamental prerequisites. The sample size was consistent with the European Federation of Psychologists’ Associations (EFPA, 2013) guidelines, and the study included participants from 60 different countries. However, a few limitations should be acknowledged. First, the GAMS used in this study had acceptable internal consistencies, except for intrinsic motivation. Our slight adjustments on a few of the items influenced the internal consistency. The reason for these adjustments was to adapt the scale for the specific Dota 2 video game context. However, removing those items did not substantially change the Cronbach’s α. Nevertheless, the GAMS scale could be further developed to accommodate different genres in video gaming. Another limitation inherent in a web-based survey as well as for any study using a self-assessment instrument is the lack of control (over, e.g., socially desirable answers), which in turn can affect the validity of the study. However, using the current recruitment strategies (i.e., web-based survey), it enabled us to attract more participants, which in turn could increase the generalizability and reliability of the results. Finally, Dota 2 is an RTS game, and the results obtained in the present study are potentially game-specific and thus may or may not be generalizable to other games or genres.

In conclusion, the present study confirms previous findings that suggest that matches played (time on task) is the strongest predictor of MMR (i.e., performance) in Dota 2. It also confirmed that perceived competence and autonomy could be factors that contribute to player performance. However, the basic psychological need relatedness, as well as motivational factors, does not predict the MMR score. The strong effect of matches played (time on task) is in line with the findings of a previous study (Röhlcke et al., 2018) and further emphasizes the effect of practice time as a critical aspect of video-game expertise.

## Data Availability Statement

The datasets generated for this study are available on request to the corresponding author.

## Ethics Statement

Ethical review and approval was not required for the study on human participants in accordance with the local legislation and institutional requirements. The patients/participants provided their written informed consent to participate in this study.

## Author Contributions

All authors have made substantial contributions to the work, revised, and made critical revisions of the manuscript. RH, MN, and BJ developed the research questions. RH, CB, and SR collected the data. RH wrote the first draft of the manuscript. RH, MN, BJ, and DS performed the formal analyses and interpretation of the data.

## Conflict of Interest

The authors declare that the research was conducted in the absence of any commercial or financial relationships that could be construed as a potential conflict of interest.

## Footnotes

1 www.who.int

2 https://www.who.int/news-room/q-a-detail/gaming-disorder
